# Design, development, and validation of a strand-specific RT-qPCR assay for GI and GII human Noroviruses

**DOI:** 10.12688/wellcomeopenres.17078.1

**Published:** 2021-09-23

**Authors:** Katja Marie Kjara König, Aminu S. Jahun, Komal Nayak, Lydia N. Drumright, Matthias Zilbauer, Ian Goodfellow, Myra Hosmillo

**Affiliations:** 1 Division of Virology, Department of Pathology, University of Cambridge, Addenbrooke's Hospital, Hills Road, Cambridge, CB2 0QQ, UK; 2 Insitute of Chemistry and Metabolomics, Center for Structural and Cell Biology in Medicine (CSCM), University of Lübeck, Lübeck, Germany; 3 Department of Paediatrics, University of Cambridge, Cambridge, UK; 4 Department of Medicine, University of Cambridge, Addenbrooke's Hospital, Cambridge, CB2 0QQ, UK; 5 Department of Medicine, University of Washington, Seattle, Washington, USA

**Keywords:** RT-qPCR, strand-specific RT-qPCR, human norovirus, calicivirus, human intestinal organoids, intestinal epithelial cells, HuNoV replicon, viral gastroenteritis

## Abstract

Human noroviruses (HuNoV) are the major cause of viral gastroenteritis worldwide. Similar to other positive-sense single-stranded RNA viruses, norovirus RNA replication requires the formation of a negative strand RNA intermediate. Methods for detecting and quantifying the viral positive or negative sense RNA in infected cells and tissues can be used as important tools in dissecting virus replication. In this study, we have established a sensitive and strand-specific Taqman-based quantitative polymerase chain reaction (qPCR) assay for both genogroups GI and GII HuNoV. This assay shows good reproducibility, has a broad dynamic range and is able to detect a diverse range of isolates. We used tagged primers containing a non-viral sequence for the reverse transcription (RT) reaction and targeted this tag in the succeeding qPCR reaction to achieve strand specificity. The specificity of the assay was confirmed by the detection of specific viral RNA strands in the presence of high levels of the opposing strands, in both RT and qPCR reactions. Finally, we further validated the assay in norovirus replicon-bearing cell lines and norovirus-infected human small intestinal organoids, in the presence or absence of small-molecule inhibitors. Overall, we have established a strand-specific qPCR assay that can be used as a reliable method to understand the molecular details of the human norovirus life cycle.

## Introduction

Human noroviruses (HuNoVs) are the leading cause of both sporadic and outbreak cases of acute gastroenteritis worldwide ^1^. Noroviruses are highly infectious, with multiple transmission routes including food sources, water, fomites and person-to-person contacts ^2^. HuNoV targets all age groups with large epidemics being frequently associated with confined settings such as long-term care facilities, restaurants, hospitals, schools and cruise ships. As a result, norovirus outbreaks result in a huge socioeconomic burden, estimated at over 60 billion dollars per year ^3^. While a number of significant steps in the understanding of norovirus gene expression and replication have been made using surrogate models such as murine norovirus (MNV), porcine sapovirus (PSaV) and feline calicivirus (FCV) ^4– 7^, recent developments have made a significant impact on the availability of tools to study norovirus biology. The HuNoV replicon system ^8, 9^ and recently established infection models including the B-cell culture system ^10^, stem cell-derived organoids ^11^ and zebrafish larvae ^12^, are all valuable norovirus experimental systems. However, there is a lack of detailed understanding of the molecular mechanisms of HuNoV replication, and many significant questions remain unanswered due to the technical limitations associated with some of these experimental systems ^13^.

**Figure 1.**
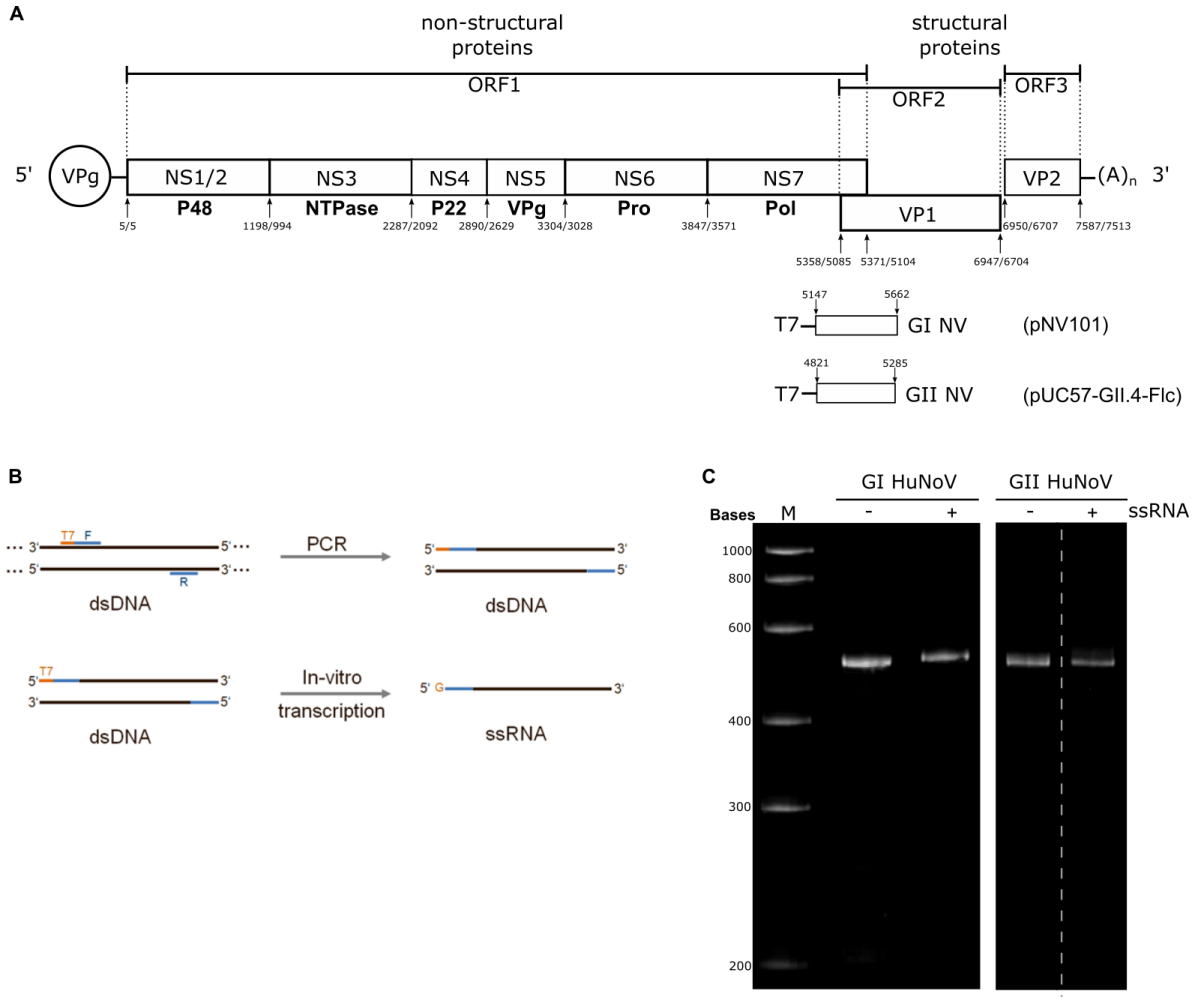
Denaturing RNA-PAGE confirms the RNA integrity of both positive and negative strands of GI and GII human norovirus. **A**) A schematic representation of the human norovirus genome showing the non-structural (NS1/2 to NS7) and structural (VP1 and VP2) proteins. The arrows indicate the position of the primer binding sites on the viral genome. GI was amplified from prototype Norwalk virus genome (Accession number M87661) whereas GII.4 was amplified from cDNA clone containing the accession number DQ658413. **B**) A schematic representation of the PCR and the *in vitro* transcriptions used to generate the strand-specific control RNAs used as standards in the strand-specific assay. **C**) The purified strand-specific RNA standards were analyzed by denaturing PAGE to confirm their integrity and size. M represents the RNA size marker in bases.

Human noroviruses have a single-stranded positive-sense RNA genome with a viral protein genome-linked (VPg) on its 5’ end. Approximately 7.4–8.3 kb in length, the genome is organized into three conserved open reading frames (ORF), 1, 2 and 3 ^14^. ORF1 is translated as a large polyprotein that encodes for the viral non-structural proteins, NS1/2 to NS7; ORF2 encodes for the major virus capsid protein, VP1; and, ORF3 encodes for the minor capsid protein, VP2 ( Figure 1A). Findings from previous studies suggest that HuNoV attaches to the cell surface using various carbohydrate attachment factors and likely enters via a yet unknown proteinaceous cellular receptor ^15– 17^. On virus entry, the VPg-linked RNA genome is released and immediately translated into viral polyprotein using the host translation machinery. The translated polyprotein is processed into immature and mature proteins which then form the viral replication complexes ^18, 19^. Within these complexes, virus replication is initiated by the synthesis of a complementary negative strand RNA, which then becomes a template for new positive strand genomic and subgenomic RNAs. RNA synthesis is thought to occur via *de novo*- and VPg-dependent mechanisms, for negative and positive sense RNA respectively ^20, 21^. The positive-sense RNA then continuously serves as a template for negative-sense RNA synthesis and *vice versa* ^21– 23^. Subsequently, the replicated genomes are packaged into the capsid, for virion assembly and exit ^4^.

Virus replication in established HuNoV replicon and culture systems is currently quantitated by RT-qPCR ^10– 12, 24^. Whilst the standard RT-qPCR assay allows for the estimation of the viral load in infected tissues, cells or stool samples ^25^, modifying the assay to enable the detection of strand-specific RNAs can be used to demonstrate active viral replication, and to better understand the molecular processes involved in the human norovirus life cycle. The development of strand-specific RNA detection and quantitation has been reported in a number of viral systems ^25– 32^. We have previously developed the strand-specific qPCR assay for MNV to study aspects of norovirus replication and to provide an additional tool to indicate that active replication is occurring ^25^. Notably, we have utilized strand-specific RT-qPCR assay to define the precise role of the stress granule assembly factor G3BP1 in the early stages of the MNV life cycle, which appears to be required prior to or at the level of viral negative sense RNA synthesis ^13^. Others have demonstrated the utility of strand-specific assays to map the dynamics of lymphocytic choriomenigitis virus replication at acute and persistent phases of infection, as well as to measure virion attachment to host cells ^32^. In alphaviruses, strand-specific qPCR assays have been used to better understand how persistent alphavirus infections are maintained in the host and to examine factors affecting the transmission cycle ^33^. For negative-sense RNA viruses such as Influenza A virus and Newcastle’s disease, strand-specific qPCR assays have been used to distinguish and quantify the three types of viral RNA (vRNA, cRNA, and mRNA) separately ^34, 35^.

Here, we have developed a sensitive and strand-specific RT-qPCR assay for HuNoV genogroups GI and GII. This assay has allowed for an accurate, precise and specific quantification of positive and negative-sense viral RNAs in replicon-containing cells and in infected human intestinal organoid-derived cultures. In both systems, we validated our assay by evaluating and analysing virus replication of GI and GII HuNoV in the presence of well characterized small molecule inhibitors targeting viral proteins (2’-C-methylcytidine and Rupintrivir), and host innate immune regulators (Ruxolitinib and Triptolide).

## Methods

### Cells and reagents

The human gastric tumour cell line harbouring the human norovirus GI replicon (HGT-NV) has been previously described ^8, 36^. Wild type HGT and HGT-NV cells were propagated in Dulbecco’s minimal essential medium (DMEM) containing 10% fetal bovine serum, 2 mM glutamine and 1% non-essential amino acids. HGT-NV cells are maintained and continuously selected in the presence of 0.75 mg/ml G418.

Primary human intestinal epithelial cells (IECs) were generated from human intestinal organoids as described ^24, 37^. In brief, intestinal biopsies were collected from human patients undergoing routine endoscopy following ethical approval (REC-12/EE/0482) and informed consent. Biopsy samples were processed immediately and intestinal epithelial organoids generated from isolated crypts following an established protocol as described previously ^24^. Following the establishment of organoid cultures, differentiated IEC monolayers were generated on collagen-coated wells in differentiation media as previously described ^24^. Confluent monolayers of differentiated IECs were then infected with HuNoV. 

Unless otherwise stated, the concentrations of the inhibitors used were as follows: 60 µM of 2’-C-methylcytidine (2-CMC, Sigma-Aldrich), 20 µM of rupintrivir (Sigma-Aldrich), 5 μM of ruxolitinib (Rux, Invivogen), and 10 nM of triptolide (TPL, Invivogen).

### Virus replication and drug treatments

HGT and HGT-NV cells were seeded on a 24-well plate with a concentration of 1.5×10 ^5^ cells/well and were treated with either 2-CMC or rupintrivir for 3 days. Three days following the initiation of treatment, the cells were lysed and total RNA was extracted using GenElute Mammalian Total RNA Kit (Sigma-Aldrich) following the manufacturer’s instructions. RNA concentrations were measured by NanoDrop spectrophotometer and normalized in nuclease-free water. Normalized RNAs were subjected to RT and strand-specific qPCR reactions described below. Methods used to analyse the data were either expressed as percentage (%) of untreated control or by fold-change.

Intestinal epithelia cells derived from human intestinal organoids were infected with either GII.3 or GII.4 HuNoV genotypes following previously published protocols ^24^. To enhance virus replication in the organoid-derived culture system, Rux or TPL were added following virus inoculation, and the drugs were maintained up to 2 days until samples were harvested. Total RNA was extracted (GenElute Mammalian Total RNA Kit, Sigma-Aldrich), and the RNA concentrations were measured by NanoDrop spectrophotometer and normalized in nuclease-free water. Normalized RNAs were subjected to RT reaction, and the effects of Rux or TPL on virus replication were evaluated by strand-specific RT-qPCR from samples collected at day 0 and day 2 post infection.

### Plasmids

pNV101 was received from Dr. Kim Green, NIH National Institute of Allergy and Infectious Disease. pNV101 is a pSPORT1 plasmid with engineered full-length cDNA clone of the Norwalk virus genome (accession numbers M87661) designated NV FL101 ^9^. pUC57-GII.4-Flc was synthesised by Biobasic and engineered to contain the complete genome of human norovirus GII.4/MD-2004/2004/US (Accession number DQ658413) under the control of a truncated T7 RNA polymerase promoter.

### Sequence alignment

Alignment of HuNoV sequences was initially carried out on the NCBI server, with sequences from GI.1 (accession number: M87661), GI.2 (accession number: L07418), GI.3 (accession number: U04469), GI.4 (accession number: AB042808), GI.6 (accession number: AF093797), GII.1 (accession number: U07611), GII.3 (accession number: U02030), GII.4 (accession number: X76716), GII.22 (accession number: AB083780), and GII.23 (accession number: KT290889). Alignments were visualised in SnapGene Viewer 5.3.1 (Insightful Science, LLC), and the relevant primer binding sites were copied and re-aligned using Clustal Omega ^38^. All genogroup and genotype designations used here are according to Chhabra *et al.* ^39^.

### Generation of dsDNA as a template for *in vitro* transcription of control material

For GI HuNoV, the dsDNA was generated by PCR using pNV101 as template, whilst for GII HuNoV, pUC57-GII.4-Flc was used as the template. PCR primers were designed with a T7 promoter sequence at the 5’ end of the forward primer of each primer pair as described in Table 1 and Table 2. The PCR reaction contained 1X KOD buffer, 0.2 mM dNTPs, 1.5 mM MgSO _4_, 0.3 µM forward primer, 0.3 µM reverse primer, 50 ng template and 1 unit of KOD in 50 µL total volume. Initial denaturation was done at 95°C for 2 min, followed by 35 PCR cycles involving denaturation at 95°C for 20 secs, annealing at 50–62°C for 15 secs and extension at 70°C for 10 secs (more details are included in Table 1 and Table 2). The final extension was carried out at 70°C for 5 min. The PCR products were purified on a 1 % agarose gel prior to use for *in vitro* transcription.

**Table 1.** List of primers used for the establishment of GI human norovirus strand-specific qPCR assay.

Primers used for the generation of standard RNAs:				
Standard RNA	Primer Name	Sequence 5’ – 3’	Position	Annealing temperature *
GI positive	T7-GIpos-F	GCGTAATACGACTCACTATAGCAAAGGAAAATACAGTTGATTTC	5147-5169	50 °C
	GIpos-R	CCATTATACATTTGTGATAGATGG	5639-5662	
GI negative	T7-GIneg-F	GCGTAATACGACTCACTATAGCCATTATACATTTGTGATAGATGG	5639-5662	50 °C
	GIneg-R	CAAAGGAAAATACAGTTGATTTC	5147-5169	
**Primers used for RT:**				
**RNA**	**Name**	**Sequence 5’ – 3’**	**Position**	
GI positive	TposGIpos	CGGGAAGGCGACTGGAGTGCCCTTAGACGCCATCATCATTYAC	5354-5375	
GI negative	TnegGIneg	GGCCGTCATGGTGGCGAATAACGYTGGATGCGNTTYCATGA	5291-5310	
**Primers used for qPCR:**				
**RNA**	**Name**	**Sequence 5’ – 3’**	**Position**	
GI positive	Tpos	CGGGAAGGCGACTGGAGTGCC	Non-viral	
	GIpos	CGYTGGATGCGNTTYCATGA	5291-5310	
GI negative	Tneg	GGCCGTCATGGTGGCGAATAA	Non-viral	
	GIneg	CTTAGACGCCATCATCATTYAC	5354-5370	
Both	GI-probe	FAM-AGATCGCGGTCTCCTGTCCA-MGBQ	5329-5348	

*Annealing temperature for generation of RNA standards

Underlined nucleotides represent the T7 promoter Shaded nucleotides represent the tag sequence

**Table 2.** List of primers used for the establishment of GII human norovirus strand-specific qPCR assay.

Primers used for generation of standard RNAs:				
Standard RNA	Primer Name	Sequence 5’ – 3’	Position	Annealing temperature *
GII positive	T7-GIIpos-F	CCGTAATACGACTCACTATAGGGACTAGGGGCCCCAACCATGAAG	4821-4844	55 °C
	GIIpos-R	GGATACTGTAAACTCTCCACCAGGG	5261-5285	
GII negative	T7-GIIneg-F	CCGTAATACGACTCACTATAGGGATACTGTAAACTCTCCACCAGGG	5261-5285	62 °C
	GIIneg-R	GGACTAGGGGCCCCAACCATGAAG	4821-4844	
**Primers used for RT:**				
**RNA**	**Name**	**Sequence 5’ – 3’**	**Position**	
GII positive	TposGIIpos	CGGGAAGGCGACTGGAGTGCCTCGACGCCATCTTCATTCAC	5081-5100	
GII negative	TnegGIIneg	GGCCGTCATGGTGGCGAATAAATGTTYAGRTGGATGAGATTCTC	5012-5034	
**Primers used for qPCR:**				
**RNA**	**Primer** **Name**	**Sequence 5’ – 3’**	**Position**	
GII positive	Tpos	CGGGAAGGCGACTGGAGTGCC	Non-viral	
	GIIpos	ATGTTYAGRTGGATGAGATTCTC	5012-5034	
GII negative	Tneg	GGCCGTCATGGTGGCGAATAA	Non-viral	
	GIIneg	TCGACGCCATCTTCATTCAC	5081-5100	
Both	GII-probe	FAM-TGGGAGGGCGATCGCAATCT -TAMRA	5048-5067

*Annealing temperature for generation of RNA standards

Underlined nucleotides represent the T7 promoter Shaded nucleotides represent the tag sequence

### *In vitro* transcription of strand-specific RNA

The strand-specific RNA standards were synthesized by *in vitro* transcription using T7 RNA polymerase. Typically, a 50 µL *in vitro* transcription reaction contained 40 mM Tris pH 8, 32 mM magnesium acetate, 40 mM DTT, 2 mM spermidine, 7.5 mM ATP, 7.5 mM CTP, 7.5 mM GTP, 7.5 mM UTP, 80 units of RNase inhibitor, 1000 ng of purified template and 150–200 units of T7 RNA polymerase. The reaction mix was incubated at 37°C for 3 h followed by a DNase I treatment (20 units) at 37°C for 30 min. The RNA was purified on a denaturing gel, visualized by UV shadowing, trizol/chloroform extracted and resolved in RNA storage buffer. The concentration of the RNA was determined by NanoDrop and Qubit flourometer, and the RNA copy numbers of each strand calculated based as the molecular mass of the ssRNA template and Avogadro’s number.

### cDNA synthesis by reverse transcription

Reverse transcription (RT) was performed using 10 ^11^ copies of either positive or negative strand RNA for the generation of the standard curve material, and 500 ng of total RNA from replicon-containing cells or organoid-derived infections. Each RNA template with the appropriate strand-specific primer flanked with a non-viral sequence tag (0.1 µM, Table 1 and Table 2) and dNTPs (0.5 mM) were combined, heated at 65°C for 5 min and incubated on ice for 5 min. The first-strand buffer (50mM Tris-HCl (pH 8.3), 75 mM KCl, 3 mM MgCl _2_), 5 mM DTT, 40 units of RNase inhibitor (RNaseOut, Invitrogen) and 200 units of Superscript III (Invitrogen) were then added. The RT reaction was performed at 55°C for 30 min and subsequently inactivated by heating at 90°C for 5 min. cDNAs were then diluted in nuclease free water (1:10) containing 4 ng/µL tRNA as carrier for the qPCR reaction.

### Strand-specific RT-qPCR assay

To generate a standard curve, cDNA templates of the positive or negative strand controls were serially diluted by 10-fold from 10 ^9^ to 10 ^2^ in the presence of 4 ng/µL tRNA as a carrier. For the strand-specific qPCR reaction, mixture of 1X PrecisionPlus qPCR MasterMix (Primerdesign), 4 µM forward primer, 4 µM reverse primer and 0.1 µM primer probe ( Table 1 and Table 2) were prepared and added to the serially diluted templates. The qPCR reaction was conducted as follows: 95°C for 10 min, followed by 45 cycles of 95°C for 15 sec and 60°C for 60 sec. Real time qPCRs were performed on a ViiA 7 real time PCR machine (Applied Biosystems, California, USA) and analysed using the ViiA ^TM^7 software v1.1 (Applied Biosystems, California, USA).

To evaluate the specificity of the assay, 10 ^8^ or 10 ^9^ copies of the opposite strand were added to each dilution of the standard curve. Then, qPCR assay was performed using the primers of the opposite strand side-by-side. To ensure the reproducibility of the assay, samples were prepared with two or three biological repeats and two technical repeats performed in more than 3 independent experiments with consistent results.

Where indicated (Extended figure 2, see Data availability statement), SYBR green-based qPCR assays were performed using 2X EGT MasterMix (Eurogentec), containing 0.2 µM forward and 0.2 µM reverse primers using identical cycling conditions to that used for Taqman-based qPCR assay.

### Statistical analysis

To demonstrate reproducibility and significance of the assay, statistical analyses were performed on duplicate or triplicate experiments using the two-tailed Student’s *t*-test (Prism 8 version 8.3.0). Figures were generated using Inkscape v0.48.1 and Prism 8 version 8.3.0.

## Results

### Generation of positive or negative RNA standards for GI and GII HuNoV

Positive and negative strands of GI and GII HuNoV RNAs were generated using the HuNoV infectious clone pNV101 ^9^ and puc57-GII.4-Flc as templates, respectively. A 517 base-pair region of GI and a 466 base-pair region of GII HuNoV at the ORF1-ORF2 junction, previously described as being highly conserved ^40, 41^, were amplified using standard PCR with the primers shown in Table 1 and Table 2. These primers were designed based on previously described diagnostic primer pairs ^9, 40^ with the addition of the T7 RNA polymerase promoter sequence to the 5’ end of the forward primer ( Figure 1B). The amplified PCR products were purified and used for *in vitro* transcription with T7 RNA polymerase. *In vitro*-transcribed RNAs were analysed by denaturing gel electrophoresis, then the RNAs were visualized by UV shadowing, excised from the gel, eluted using the crush and soak method, and purified by phenol/chloroform extraction. To ensure the accurate quantification of the purified RNAs the concentrations were determined by Nanodrop and Qubit flourometer. Aliquots of the purified RNAs were examined on a denaturing PAGE to confirm the integrity of the RNA. Purified strand-specific control RNAs showed a single species of RNA of about 500 bases ( Figure 1C). Each of the RNA standards for both the positive and negative sense RNAs were then diluted and stored at a concentration of 10 ^11 ^copies/µL for subsequent procedures.

### Establishment of a strand-specific RT-qPCR assay using tagged RT primers

The accurate quantification of specific viral RNA strands can be hindered by false priming during reverse transcription. Such false priming has been previously observed in assays developed for MNV and a number of other RNA viruses, including HCV, foot-and-mouth disease virus (FMDV), influenza virus, dengue virus, alphaviruses, and rhabdovirus ^25, 27– 29, 31, 34, 42^. Modified RT-primers containing a non-viral tag sequence, along with virus-specific sequences, were designed with the aim of generating tagged cDNA less prone to subsequent false priming. For the subsequent qPCR, one of the amplification primers was designed to target the non-viral tag sequence only and was combined with a single virus-specific primer to allow the specific amplification of the tagged viral cDNA only. This approach has been previously used to improve the specificity of quantification ^25, 33^.

Using this approach, we designed strand-specific RT primers, TposGIpos and TnegGIneg for GI HuNoV, and TposGIIpos and TnegGIIneg for GII HuNoV, to generate cDNA from either the positive or negative strand of viral RNA respectively. In each case non-viral tag sequences were added to the 5’ end as described in Table 1 and Table 2. A Taqman-based qPCR assay was designed using the primer pairs consisting of the tag-specific (Tpos or Tneg) and virus-specific primer for the positive strand (GIpos or GIIpos) or negative strand viral RNA (GIneg, GIIneg), which was combined with virus probes specific for GI or GII HuNoV ( Table 1 and Table 2). The non-tag parts of the RT primers, qPCR primers and probes bind highly conserved regions of the HuNoV genome (Extended figure 1, see Data availability statement), and were adapted from primer/probe combinations previously described by Kagayama *et al.* ^40^.

**Figure 2.**
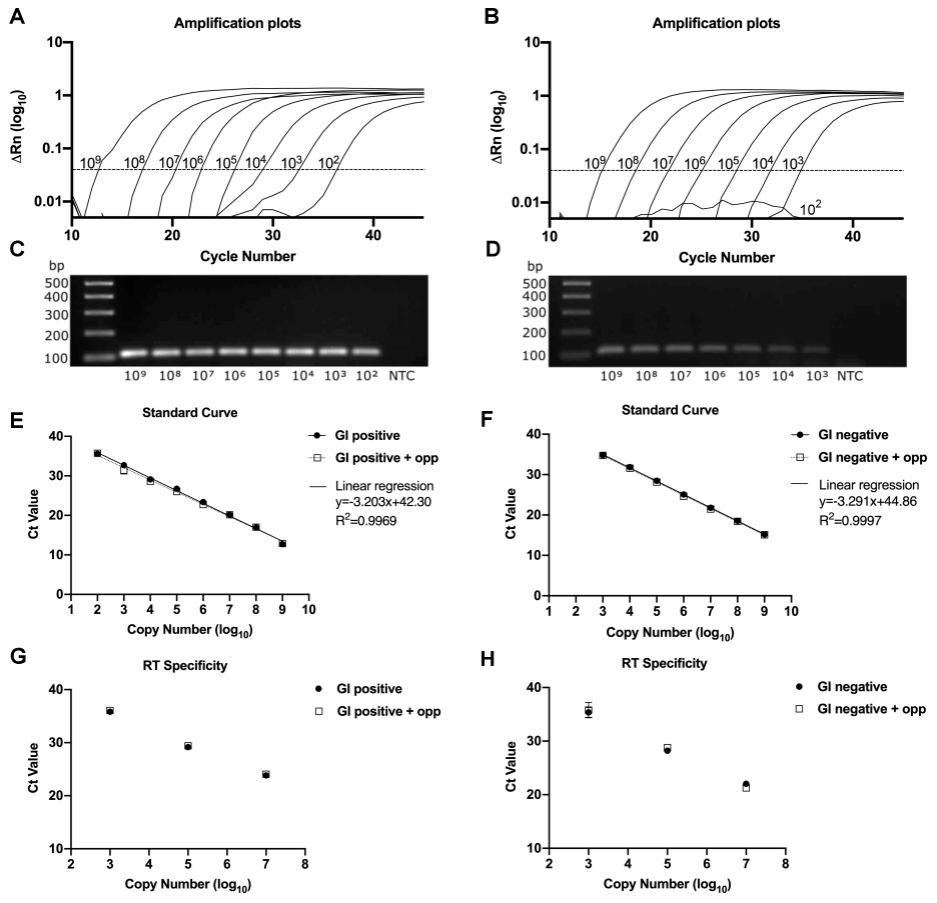
Evaluation of the real-time strand-specific qPCR assay for HuNoV Genogroup I. The left-hand column represents positive strand and the right-hand column represents negative strand. **A**, **B**) Strand specific qPCR amplification plots for positive and negative sense GI viral RNA plotted against the cycle number. The standard curve represents 8 or 7-point ten-fold dilutions from 10 ^9^ copy numbers. **C**, **D**) Confirmation of strand-specific qPCR products and no template control (NTC) by agarose gel electrophoresis. **E**, **F**) Strand specific qPCR assay was performed and plotted in presence or absence of a fixed amount of the opposite strand. Solid circle in line ( 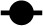) represents linear regression of the standard curve; square symbol in dotted line ( 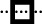) indicates the linear regression in the presence of opposite strand (+opp). Slope (y) and regression coefficient (R ^2^) of the standard curve were shown. **G**, **H**) To mimic an infection condition, the RT reactions were performed in the presence of high copy number of the opposite strand. Solid circle ( 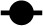) represents 10 ^7^, 10 ^5^ and 10 ^3^ copies of the standard RNA with 10 ^6 ^copies of the opposite strand (+opp) in square symbol ( 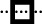). Samples were analyzed in three biological replicates and two technical replicates and the results confirmed in more than 3 independent experiments. Each point was plotted as mean ± SD.

To validate the strand-specific qPCR assay, we then examined the linearity and sensitivity of serial dilutions down to 100 genome copies per reaction. The standard curves of either positive or negative strands for GI HuNoV produced a linear response across 8 points of a 10-fold dilution series, with a correlation coefficient (R ^2^) of 0.9969/0.9997 and a slope of -3.2/-3.3 for the positive and negative strand, respectively ( Figures 2E and 2F). This corresponded to a detection limit of ~100 copies for positive and ~1000 copies for the negative strand ( Figures 2A and 2B). The amplified products were also visualized by gel electrophoresis and confirmed as a single product corresponding to the expected size (106 bp) ( Figures 2C and 2D). To evaluate the specificity of the assay, we examined the impact of including high levels of the opposite strand in the reaction, as well as using primer pairs designed to detect the opposite strand. The presence of 10 ^8^ copies of the opposite strand during the qPCR or 10 ^10^ copies during the RT reaction, did not affect the linearity or sensitivity of the assay, confirming the specificity of the strand specific RT-qPCR assay ( Figures 2E, 2F, 2G and 2H).

To improve the sensitivity for GI negative strand ssqPCR assay, we also developed a SYBR green-based qPCR assay. We found that the SYBR-based assay provided a lower detection limit, allowing for as few as 100 genome copies to be reproducibly detected, with slope of -3.1 and R ^2^ of 0.9946 (Extended figure 2A-C, see Data availability statement). The presence of 10 ^8^ copies of the opposite strand during the qPCR showed a similar linearity as the standard confirming the assay specificity for GI HuNoV strand-specific qPCR (Extended figure 2C, see Data availability statement). Thus, modification of the strand-specific qPCR assay for GI negative strand using a SYBR-based protocol was able increase the sensitivity of detection down to 100 genome copies per reaction.

**Figure 3.**
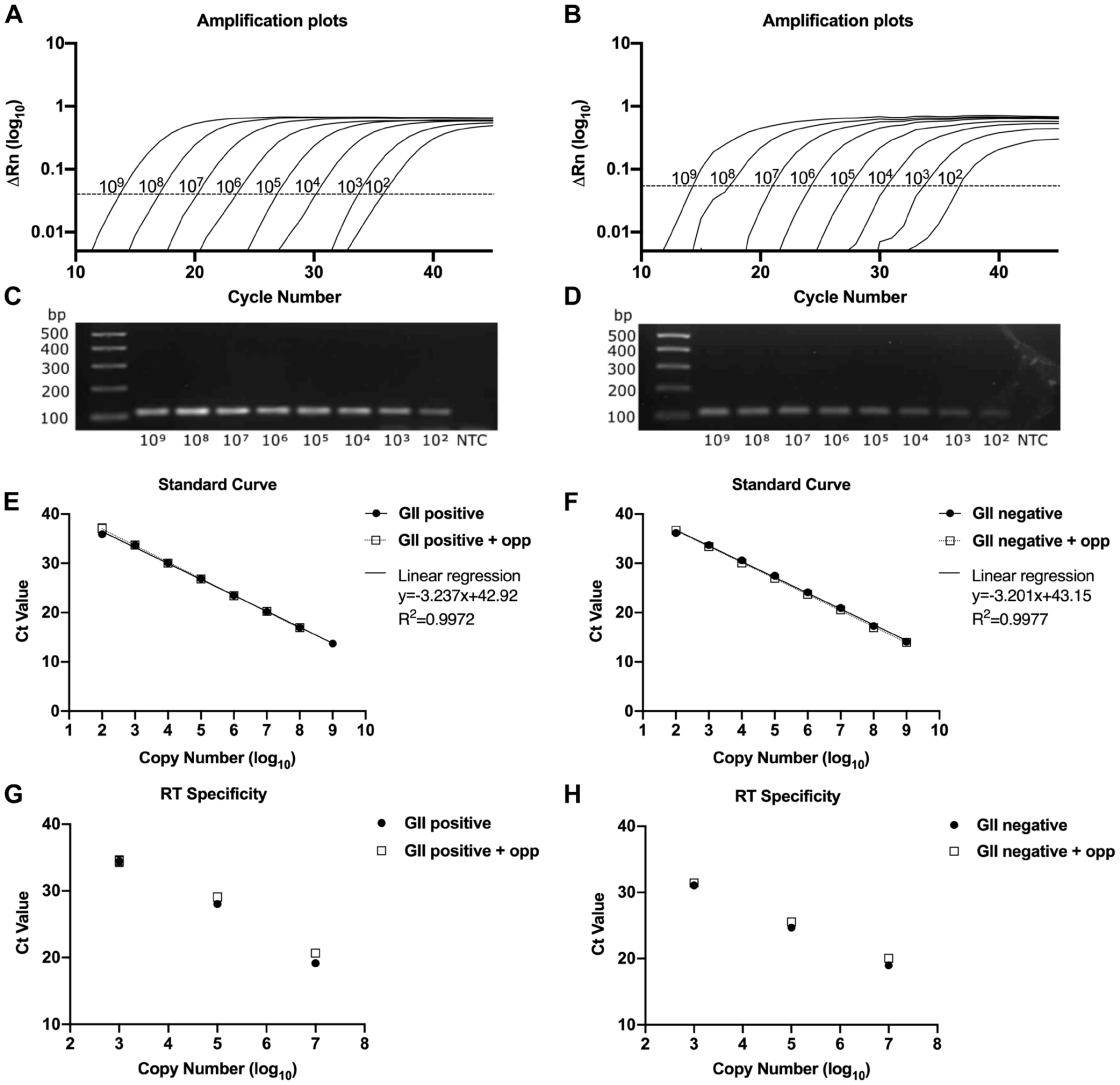
Evaluation of the real time strand-specific qPCR assay for HuNoV Genogroup II. The left-hand column represents positive strand and the right-hand column represents negative strand. **A**, **B**) Strand-specific qPCR amplification plots for positive and negative sense GII viral RNA plotted against the cycle number. The standard curve represents 8-point 10-fold dilutions from 10 ^9^ to 10 ^2 ^copy numbers. **C**, **D**) Confirmation of strand-specific qPCR products and no template control (NTC) by agarose gel electrophoresis. **E**, **F**) Strand-specific qPCR assay was performed and plotted in presence or absence of a fixed amount of the opposite strand. Solid circle in line ( 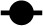) represents linear regression of the standard curve; square symbol in dotted line ( 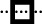) indicates the linear regression in the presence of opposite strand (+opp). Slope (y) and regression coefficient (R ^2^) of the standard curve were shown. **G**, **H**) To mimic infection conditions, RT reaction was analysed in the presence of high copy number of the opposing strand. Solid circle ( 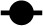) represents 10 ^7^, 10 ^5^ and 10 ^3^ copies of the standard RNA with 10 ^6^copies of the opposite strand (+opp) in square symbol ( 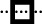). Samples were analysed in three biological replicates with two technical replicates and performed in more than 3 independent experiments. Each point was plotted as mean ± SD.

Similarly, the GII HuNoV standard curves of either the positive or negative strands displayed a linear response across 8 points of a 10-fold dilution series, with a correlation coefficient (R ^2^) of 0.9972/0.9977 and a slope of -3.2 for positive and negative strand respectively ( Figures 3E and 3F). In this instance, both strand-specific qPCR assays had a detection limit of 100 genome copies per reaction ( Figures 3A and 3B). The presence of 10 ^9^ copies of the opposite strand during the qPCR or 10 ^10^ during the RT also showed a similar linearity as the standard reference demonstrating strand specificity of the GII HuNoV qPCR assay and the RT reaction ( Figures 3E, 3F, 3G and 3H). The intra-assay reproducibility of the strand-specific qPCR assays for both GI and GII HuNoV consistently showed that both biological and technical replicates align similarly in the curve within an acceptable standard deviation ( Figure 2 and Figure 3). Furthermore, when the expected and detected Ct values obtained using established strand-specific RT-qPCR assay along with conditions that mimicked viral infections were compared, the corresponding Ct values in each determined copy numbers were similar ( Table 3).

**Table 3.** Comparison of the expected and detected Ct values obtained using the established strand-specific RT-qPCR assay, using conditions that mimicked viral infection. cDNAs were synthesized using serially diluted *in vitro* transcribed RNA in the presence of 10 ^10^ copies of the opposite strand (+opp).

RNA	Copy number	Expected Ct value	Detected Ct value
GI positive + opp	10 ^7^ 10 ^5^ 10 ^3^	23.8 29.2 35.8	24.1 29.4 36.1
GI negative + opp	10 ^7^ 10 ^5^ 10 ^3^	22.0 28.2 35.4	21.3 28.8 35.8
GII positive + opp	10 ^7^ 10 ^5^ 10 ^3^	19.2 28.0 34.5	20.7 29.1 34.5
GII negative + opp	10 ^7^ 10 ^5^ 10 ^3^	19.0 24.7 31.1	20.1 25.5 31.4

### Absolute quantification of viral positive- and negative-sense RNAs contained in GI HuNoV replicon-bearing cells

**Figure 4.**
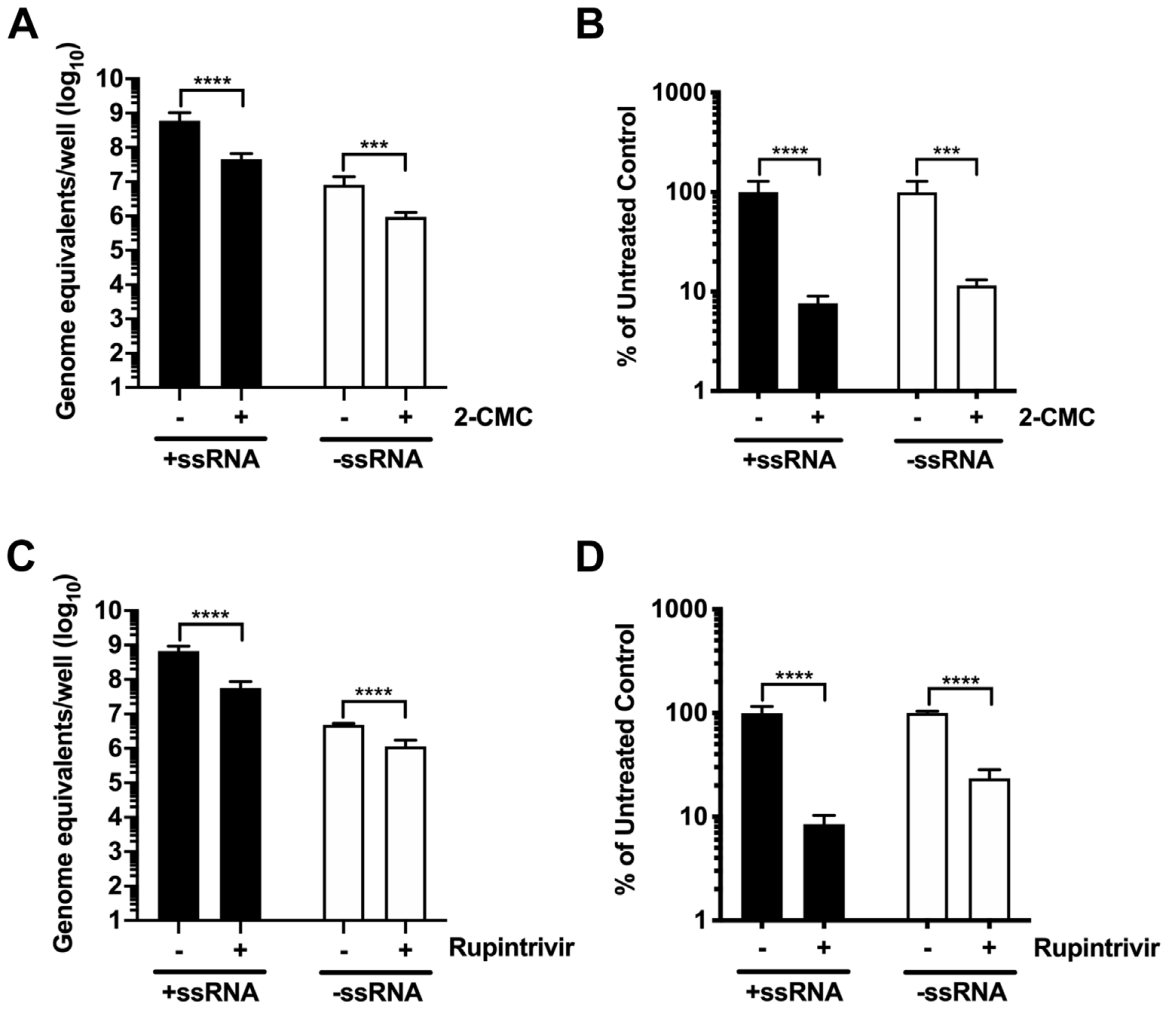
HuNoV inhibitors 2'-C-methylcytidine (2-CMC) and rupintrivir reduce both the positive and the negative strand of GI HuNoV. The impact of 2-CMC and rupintrivir on HuNoV replication was examined in HGT-NV cells. Cells treated with and without 2-CMC or rupintrivir were harvested after 3 days. Extracted RNAs were quantified and analyzed by strand-specific RT-qPCR. **A**, **C**) The levels of positive (+ss) and negative (-ss) RNA strands in the absence or presence of 2-CMC ( **A**) and rupintrivir ( **C**) were measured as genome equivalents/well. **B**, **D**) The effect of treatment on virus replication was plotted as percentage of untreated control. The mean ± SEM from triplicate samples analyzed in technical duplicates is plotted. Statistically significant values are represented as: ***=p≤0.001, ****=p≤0.0001.

Having established the strand-specific qPCR assay for GI HuNoV, we confirmed that it could be used to assess the effect of inhibitors on GI HuNoV replication. For this we examined the impact of the RNA polymerase inhibitor 2-CMC and the protease inhibitor rupintrivir, on viral RNA synthesis in GI HuNoV replicon containing cells (HGT-NV cells). 2-CMC is a well-characterized nucleoside analogue that effectively targets the HuNoV viral polymerase, thereby inhibiting the production of viral RNA ^43, 44^. We treated HGT and HGT-NV cells with 0 and 60 µM concentrations of 2-CMC and measured the levels of positive and negative RNA strands. As expected, the levels of positive strand RNA was ~100 fold higher than the negative strand, as previously reported for other noroviruses ^25^. Treatment of GI replicon-containing cells with 2-CMC resulted a reduction of viral RNA levels by 93% for positive strands and 88% for the negative strands by day three post treatment ( Figures 4A and 4B), confirming the negative effect of 2-CMC in human norovirus RNA synthesis.

Rupintrivir, an irreversible inhibitor of the human rhinovirus 3C protease, has also been reported to inhibit the replication of the Norwalk virus replicon ^45^. Recently, characterisation of rupintrivir in HGT-NV cells has identified amino acid substitutions in the viral protease that are necessary for proteolytic processing of the polyprotein ^36^. We found that treatment of GI replicon-containing cells with rupintrivir resulted in a 92% and 77% reduction in viral positive and negative strand RNA, respectively, after three days post treatment ( Figures 4C and 4D).

### Determination of positive- and negative-sense viral RNAs in GII HuNoV-infected organoid-derived cultures

**Figure 5.**
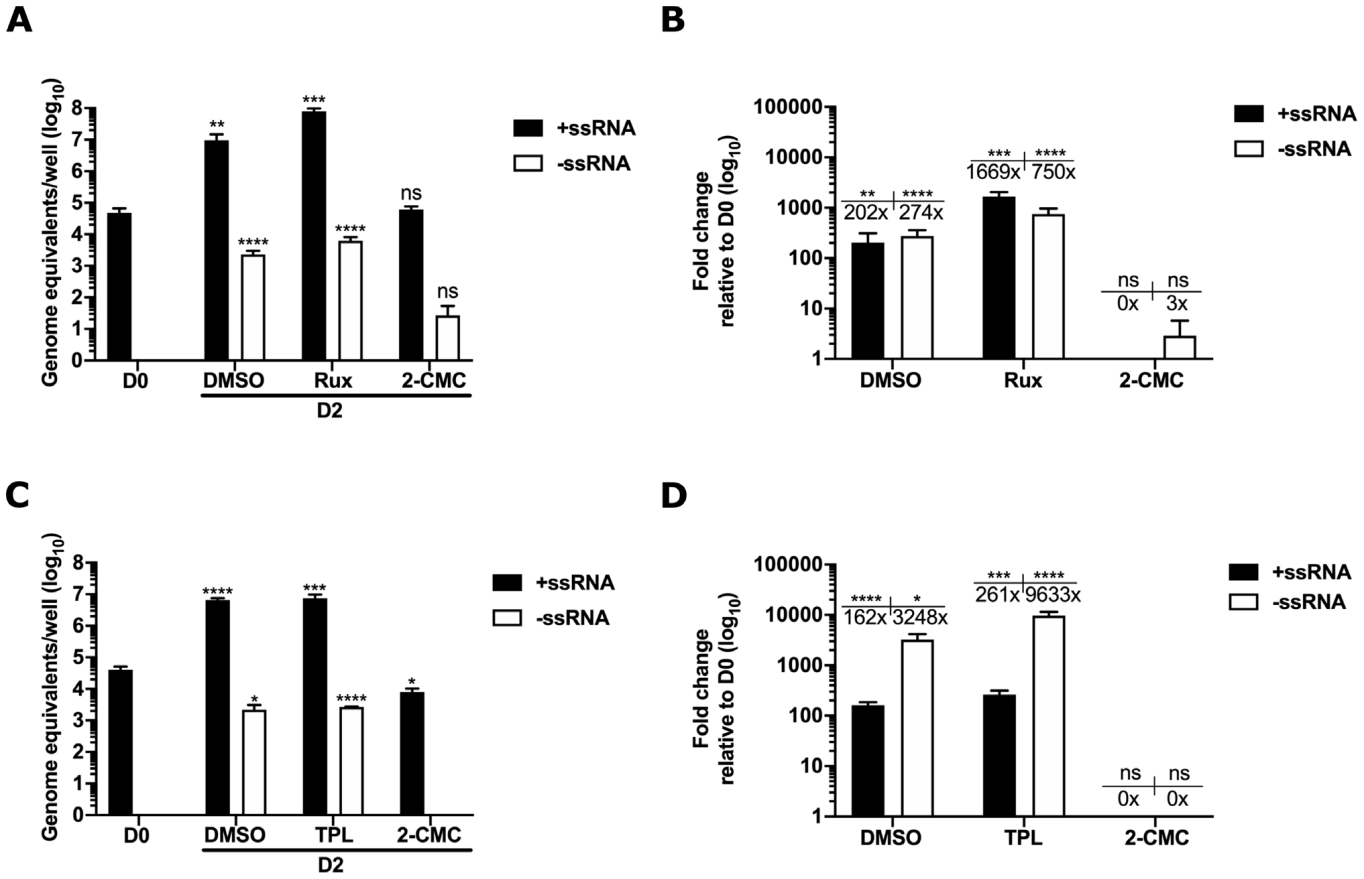
Innate immune suppressors ruxolitinib (Rux) and triptolide (TPL) increase both the positive and the negative viral RNA strands in GII HuNoV infected organoid-derived culture system. Intestinal epithelial cells-derived from human duodenal organoids were inoculated with GII.3 HuNoV and ileal organoids with GII.4 HuNoV then, infected cells were subsequently treated with either DMSO, Rux, TPL or 2-CMC, as relevant control. The effects of innate immune inhibitors on HuNoV replication were examined after 2 days. The RNA extracts collected on day 0 (D0) and day 2 (D2) post infection were quantified and analyzed by strand-specific RT-qPCR. ( **A**, **C**) The levels of positive (+ss) and negative (-ss) RNA strands in the absence or presence of the drug/s were measured as genome equivalents/well. ( **B**, **D**) The effect of treatments on virus replication was plotted as fold change relative to D0. The mean ± SEM from triplicate samples analyzed in technical duplicates is plotted. Statistically significant values are represented as: ns=p>0.05, *=p≤0.05, **=p≤0.01, ***=p≤0.001, ****=p≤0.0001.

The use of intestinal epithelial cells (IECs) derived from human intestinal organoids is pivotal in establishing a robust HuNoV culture system ^11^. This breakthrough has opened opportunities to better understand molecular mechanisms of viral RNA replication in HuNoV-infected cells. Recently, we demonstrated that replication of HuNoV in IECs results in interferon-induced transcriptional responses and that HuNoV replication in IECs is restricted by the interferon response ^24^. The modulation of this response through treatment with small-molecule inhibitors of components of the interferon pathway enhances HuNoV replication in IECs ^24^. To confirm the utility of the GII HuNoV strand-specific assay in HuNoV-infected organoid-derived cultures, we initially examined the effect of an inhibitor (Ruxolitinib, Rux) that specifically targets Janus kinases JAK1/JAK2, on the levels of viral RNA in IECs. JAK1/2 are involved in an early stage of interferon signalling and are activated following engagement of interferons with their cell surface receptor. We also included 2-CMC as a known inhibitor of the norovirus RNA polymerase. In the absence of Rux, we observed that the levels of both positive and negative strands increased by 202 and 274 fold, respectively, over the two day infection of IECs with GII.4 HuNoV ( Figures 5A and 5B). No negative strand viral RNA was observed at D0 as expected ( Figure 5A), likely due to the high specificity of viral genome packaging during the production of infectious virions. In the presence of Rux, we observed a 1669-fold increase in positive-sense RNA and a 750-fold increase in negative-sense RNA, confirming our previous observation that the inhibition of interferon signalling resulted in a significant improvement in HuNoV replication in IECs ^24^.

We next examined the effect of TPL, a compound extracted from a traditional medicinal plant ( *Tripterygium wilfordii* Hook F), exhibiting a broad pharmacological effects against inflammation, fibrosis, cancer, viral infection, oxidative stress and osteoporosis ^46, 47^. TPL is reported to modulate the activity of many genes including those involved in apoptosis and NF-kB-mediated responses and has recently been shown to selectively impair RNA polymerase II activity ^48, 49^. To explore the impact of TPL on HuNoV replication, IECs were inoculated with HuNoV GII.4 strain, then treated with either DMSO as a control, TPL or 2-CMC ( Figures 5C and 5D). In the absence of TPL, we observed robust virus replication with 162- and 3248-fold increases in the positive and negative strands, respectively ( Figure 5D). However, the addition of TPL enhanced replication, leading to a 261-fold increase in positive-sense and 9633-fold increase in the negative-sense RNA. 2-CMC inhibited HuNoV replication as expected. Altogether, our findings here consistently agree with the previous observations ^24^ and strengthens the hypothesis that HuNoV replication is inhibited by TPL-sensitive IFN responses.

## Discussion

The presence of full-length negative strand viral genomic RNA is a hallmark of RNA virus replication within an infected cell or tissue. As such, methods for detecting and quantifying specific strands of viral RNA are important in the study of RNA viruses. As previously noted, unlike the standard qPCR assays, the development of strand-specific qPCR can be challenging due to false priming ^25– 27, 33, 34^, but the challenges associated with generating control RNAs that contain only the strand of interest have been overcomed using various strategies. In this study, we established sensitive and specific RT-qPCR assays for both GI and GII HuNoVs. The strand-specific assays developed here were generated by modifying the most widely used diagnostic RT-qPCR assays ^40, 41^. Noroviruses are a highly diverse group of viruses with genogroup I being split into at least 9 genotypes and genogroup II having at least 27 different genotypes ^14^. These assays target one of the most conserved regions of the HuNoV genome, namely the ORF1-2 junction, and based on an alignment of available sequences, we predict that they are able to detect multiple genotypes within each genogroup.

To develop the strand-specific assay, we employed the use of tagged RT primers that contain non-viral sequences at the 5′ end of a viral strand-specific sequence, allowing us to achieve specificity even in the presence of high levels of the opposite strand in either the qPCR reaction or during cDNA synthesis ( Figure 2 and Figure 3). While the detection limit of the GI HuNoV negative sense RNA was ~1000 copies per reaction, modifying the assay to use SYBR chemistry improved the sensitivity to as low as ~100 copy numbers. Finally, we have applied and validated our strand-specific qPCR assay in the presence of on-going virus replication using either replicon-containing cells or organoid-derived cultures ( Figure 4 and Figure 5). Another key factor to the development of the assay was the ability to generate robust strand-specific control RNAs to act as standards. T7 RNA polymerase frequently extends the 3’ end of RNAs via *cis* self-primed extension, whereby the product RNA rebinds to the polymerase and self-primes (in *cis*) generation of a hairpin duplex ^50^, resulting in a double-stranded RNA product. The presence of this dsRNA product compromises the specificity of the assay, therefore we utilised denaturing PAGE to gel purify only single-stranded RNA of uniform length. RNA templates prepared in this way generated pure, intact, strand-specific RNAs as standard references.

It is well recognized in the calicivirus field that the culture systems currently available for HuNoV have technical limitations ^51, 52^ and as such lack the ability to accurately and efficiently assess the presence of infectious virus e.g. via a standard tissue culture infectious dose 50 (TCID50) determination or plaque assay. As a result, RT-qPCR has become the method of choice when assessing HuNoV replication. This GI and GII strand-specific assay was found to be suitable for the characterisation of HuNoV replication in cell culture. Selected drugs such as 2-CMC and rupintrivir significantly inhibited GI HuNoV replication resulting in a decrease in both RNA strands, confirming their effects in suppressing HuNoV RNA synthesis and proteolytic processing, respectively ( Figure 4). We were also able to clearly detect the presence of the viral negative-sense RNA during GII.4 HuNoV replication in IECs over a 2-day period ( Figure 5). Treatment with the innate immune inhibitors such as ruxolitinib and triptolide validated the previously observed enhancement in virus replication in organoid-derived cultures ( Figure 5).

Overall, the strand-specific assay described here provides a valuable tool with which to examine aspects of the human norovirus life cycle. We have demonstrated its utility to detect active norovirus replication in culture via the robust detection of viral negative strand RNA and to examine the impact of various inhibitors on the viral life cycle. This strand-specific assay therefore is a novel and useful tool with which to uncover the role of cellular proteins and pathways in the HuNoV life cycle.

## Data availability

Apollo. *Research data supporting "Design, development, and validation of a strand-specific RT-qPCR assay for GI and GII human Noroviruses"* DOI: https://doi.org/10.17863/CAM.74820 ^53^

This project contains the following data:

- 1. raw images collected directly gel-doc imager 2. raw qPCR data files exported from Viia7 or StepOnePlus qPCR machine 3. sequence alignment files for calivirus strains presented in the manuscript

Data are available under the terms of the Creative Commons Attribution 4.0 International license (CC-BY 4.0).
