# Supplementary material for: Design, development, and validation of a strand-specific RT-qPCR assay for GI and GII human Noroviruses
Source: Wellcome Open Res. 2021 Sep 23;6:245. [Version 1] doi: 10.12688/wellcomeopenres.17078.1 (PMC8506223; doi:10.12688/wellcomeopenres.17078.1)
Supplement: Peer review history

# Peer review: Reviewer response for version 1

**DOI:** 10.21956/wellcomeopenres.18865.r46115

**Published:** 2021-10-14

**Authors:** Sanghyun Lee

**Affiliations:** 1 Department of Molecular Microbiology and Immunology, Brown University, Providence, RI, USA

In this article, Konig and colleagues provide an important methodology that detects negative- or positive-strand genomic RNA without signal crossover. Given the absence of robust culture models for human noroviruses, assessment of norovirus life cycle has been very difficult. Quantitative RT-PCR is a widely used method to diagnose norovirus and to measure norovirus replication in cells. The same author group has provided a similar qPCR technique for murine norovirus, which has been used in multiple studies in the field. In this study, they expanded their expertise to detect strand-specific viral RNA genomes for human noroviruses. Two tested assays for noroviruses, GI.I and GII.4, exhibited high sensitivity and specificity. One of the exciting parts of this study is that they applied the technique to the amplicon culture and the organoid culture models with the treatment of viral inhibitors. This study is well-designed and well-written and has a protocol that can be reproduced in many other norovirus labs in the field. 

**Major comments:**

1. The information about GII.4 human norovirus inoculum is not described in the Methods section, in terms of source, preparation, and titer/dose of the GII.4 virus. 
2. Figure 3C and 3D were not mentioned throughout the manuscript. All figures should be described at least one time in the manuscript. 
3. In Figures 5B and 5D, adjusting the order of panels would be helpful to draw the conclusion easier. For example, “DMSO+ssRNA Rux+ssRNA, 2-CMC+ssRNA; DMSO-ssRNA Rux-ssRNA, 2-CMC-ssRNA” would make the figure straightforward to readers.

Is the rationale for developing the new method (or application) clearly explained?

Yes

Is the description of the method technically sound?

Yes

Are the conclusions about the method and its performance adequately supported by the findings presented in the article?

Yes

If any results are presented, are all the source data underlying the results available to ensure full reproducibility?

Yes

Are sufficient details provided to allow replication of the method development and its use by others?

Yes

Reviewer Expertise:

Virology and Immunology

I confirm that I have read this submission and believe that I have an appropriate level of expertise to confirm that it is of an acceptable scientific standard.

# Peer review: Reviewer response for version 1

**DOI:** 10.21956/wellcomeopenres.18865.r46112

**Published:** 2021-10-11

**Authors:** Morgan Herod

**Affiliations:** 1 School of Molecular and Cellular Biology, Faculty of Biological Sciences, Astbury Centre for Structural Molecular Biology, University of Leeds, Leeds, UK

This article describes the design and development of a strand-specific quantitative PCR against genogroups I and II human noroviruses. The authors optimise the methodology using *in vitro* produced controls, achieving good specificity and sensitivity for both genomic and anti-genomic strands, before applying the methodology to quantify RNA in a norovirus replicon cell line and norovirus-infected human organoid cultures. As explained by the authors, the described method will be useful for studying fundamental mechanisms of human norovirus replication using e.g. human organoid cultures, where other measurements of viral replication are not possible. The methods are described in detail to make them easily reproducible and the results and conclusions accurately presented. The study generates several interesting questions that would be good to investigate, such as:

- Can the authors use this technology to look at the temporal regulation/separate of positive- and negative-synthesis synthesis or for example the half-life of RNA strands?
- The relative reduction in negative strands appears to be less than for positive strands upon inhibition (see for example data in Figure 4). Is this genuine and reflective of the relative ratio of positive/negative strands or is something else going on here?

One piece of data that was not clear, in figure 5A and B the authors calculate a 274-fold increase in negative strand synthesis in day 2 compared to day 0 (as described in the text), however, there are no negative strands at day 0. How did the authors calculate a fold change over zero?

Is the rationale for developing the new method (or application) clearly explained?

Yes

Is the description of the method technically sound?

Yes

Are the conclusions about the method and its performance adequately supported by the findings presented in the article?

Yes

If any results are presented, are all the source data underlying the results available to ensure full reproducibility?

Yes

Are sufficient details provided to allow replication of the method development and its use by others?

Yes

Reviewer Expertise:

Virology

I confirm that I have read this submission and believe that I have an appropriate level of expertise to confirm that it is of an acceptable scientific standard.

# Peer review: Reviewer response for version 1

**DOI:** 10.21956/wellcomeopenres.18865.r46113

**Published:** 2021-09-30

**Authors:** Robyn Hall

**Affiliations:** 1 Health & Biosecurity, Commonwealth Scientific and Industrial Research Organisation (CSIRO), Acton, ACT, Australia

In this study, König *et al.* report a method for detecting positive and negative sense norovirus RNAs using a strand-specific two-step RT-qPCR method. They develop and validate two assays, one for GI noroviruses and one for GII noroviruses, using genogroup-specific *in vitro* transcripts as standards for quantification. Briefly, template RNAs are reverse-transcribed using genogroup-specific gene-specific primers for RT, which are tagged with a non-viral sequence. cDNAs are then subjected to Taqman or SYBR-based qPCR using primers targeting the non-viral tag and conserved viral sequences. The assays for both genogroups, for both +ssRNA and -ssRNA are shown to be specific (for the sense of interest) and sensitive. They then use this assay to quantify positive and negative sense RNAs using both a norovirus replicon system and infectious norovirus in an intestinal organoid system.

 The innovative methods developed in this paper, such as the use of a non-viral tag, overcome known limitations previously associated with strand-specific PCR. This is a clear and convincing demonstration of the utility of this method for robustly quantifying viral replication, with many advantages over alternative methods such as using Northern blots. In my opinion, the authors have appropriately validated their methods and have provided sufficient detail for these assays to be implemented by other research groups. This manuscript was well-written, followed a logical progression, and was very nice to read.

 I have several minor suggestions that may help to further improve the manuscript.

**Introduction**:

 Figure 1 states that the arrows represent primer binding sites, however, the arrows along the full genome schematic seem to represent protease cleavage sites. In fact, I would suggest splitting this figure into that showing the genome schematic and then a new figure showing the method set up and PAGE as the first figure of the results, since these are new results being presented in current 1B and 1C. Additionally, I suggest clarifying in the figure legend “GI was amplified from a full-length Norwalk virus cDNA clone (accession M87661), whereas GII.4 was amplified from the full-length GII.4/MD-2004/2004/US pUC57-GII.4-Flc cDNA clone (accession DQ658413)”.

 I think this figure would benefit from showing the whole workflow for both positive and negative sense RNAs, from the PCR and in vitro transcription steps (as already shown) plus the RT and qPCR steps. This would be helpful for showing each of the primer binding sites from Tables 1 and 2 and the different orientations of the +ssRNA and -ssRNA workflow at each step. 

 “Newcastle’s disease” should read “Newcastle disease virus”.

**Methods**:

 Virus replication and drug treatments: Have the GII.3 and GII.4 viruses used in this study been sequenced and do they have accession numbers? I would suggest giving the full genotype (pol P-type and VP60 type) for completeness, since different variants may have different biological characteristics.

 Sequence alignment: What is meant by relevant primer binding sites? Can this wording be clarified?

 cDNA synthesis:

 For clarity, it may be helpful to specify “positive or negative strain RNA from in vitro transcription reactions” instead of simply “positive or negative strand RNA”.

 Similarly, I suggest clarifying “appropriate” e.g. genogroup-specific.

 Typically SSIII is heat-inactivated at 70C for 15 minutes, is there a reason this was modified to 90C for 5 min?

 Strand-specific RT-qPCR assay: It may be helpful to further clarify what is meant by “specificity” e.g. strand specificity. It could also be useful to be explicit here about what was done. I found that the multiple use of ‘opposite’ made it hard to follow the switches. I suggest something like “To evaluate the specificity of the assay, 10 ^8^ or 10 ^9^ copies of the opposite strand were added to each dilution of the standard curve. For example, 10 ^8 ^copies ^­^ of negative strand cDNA was added to each dilution of the positive strand cDNA standard curve and qPCR was performed with the positive strand qPCR assay, and vice versa. Figures 2E&F and 3E&F should be referenced here (I think?).

**Results**:

 Generation of positive or negative RNA standards for GI and GII HuNoV: I suggest explicitly using “ *in vitro *transcripts” instead of “strands”.

 Figures 2 & 3:

 I suggest stating the “fixed amount” in the figure legend. Is this the 10 ^8 ^or 10 ^9 ^from the methods section? This really highlights that even in the presence of overwhelming ratios of opposite sense to target sense template there is no loss of specificity!

 I found E&F a little unclear – is e.g. ‘negative’ vs ‘negative + opp’ simply without and with the “fixed amount of the opposite strand” spiked in? And since the Cts don’t shift at all this highlights that there is no amplification of the non-target strand?

 Table 3:

 Where does the 10 ^10 ^amount come from? Various amounts are mentioned through the manuscript from 10 ^8^, 10 ^9^, 10 ^10^, 10 ^6^, and ‘fixed’ and I found it hard to follow what context these different amounts were used in.

 It would be helpful to clarify what is meant by ‘expected Ct value’, both here and in the relevant results section. Is this shown to demonstrate that there is no non-target strand amplification and also no inhibition from the addition of the non-target strand? Is table 3 necessary when this is already demonstrated in Figs 2&3 E – H?

 I am interested in the methodology of deriving genome equivalents per well when the RNA was normalised to a standard concentration as described in the methods. Would it not be simpler to extract from the contents of the entire well, elute in say 50 μl, quantify using 1 μl, and then multiply the result by 50 to derive the per well units?

 Figure 5:

 Were the inoculums used here stool filtrates? Would it not be expected for there to be some -ssRNA in these samples as well, since presumably actively replicating virus would have been present? Or do you assume only virions are present in positive stool samples, and not shed infected cells containing -ssRNA? Or is it assumed that any infected cells would lyse and free RNAs would be degraded prior to RNA extraction?

 I would find it easier to group the +ssRNA and -ssRNA together and colour/pattern by treatment, so that it is clear that the change being measured is relative to the same strand at D0.

 I find it curious that the increase in -ssRNA for 2-CMC in A is not significant, yet the decrease in +ssRNA in C is. It’s also interesting that in C, TPL +ssRNA was higher (261x) than DMSO (162x) yet only 3* compared to 4*. Is this because of the differences in errors?

 Similarly, TPL and DMSO -ssRNA were similar levels but very different significance levels. Is this again due to the size of the error bars?

 In D, 2-CMC +ssRNA is marked as 0x change but in C it is clearly shown to significantly decrease – this should be shown as a fold decrease in D, or the plots should be adjusted to ‘fold increase’ rather than fold change (although I still feel fold change is more appropriate).

 Were uninfected control cells included?

 I really appreciated that the raw data files were made accessible! This is fantastic to see.

Is the rationale for developing the new method (or application) clearly explained?

Yes

Is the description of the method technically sound?

Yes

Are the conclusions about the method and its performance adequately supported by the findings presented in the article?

Yes

If any results are presented, are all the source data underlying the results available to ensure full reproducibility?

Yes

Are sufficient details provided to allow replication of the method development and its use by others?

Yes

Reviewer Expertise:

veterinary virology, infectious disease epidemiology, caliciviruses, host-pathogen coevolution, genomic epidemiology and evolution, diagnostic test development, metatranscriptomics, lagomorph diseases

I confirm that I have read this submission and believe that I have an appropriate level of expertise to confirm that it is of an acceptable scientific standard.
